# Controlling deposition of nanoparticles by tuning surface charge of SiO_2_ by surface modifications†
†Electronic supplementary information (ESI) available. See DOI: 10.1039/c6ra22412a



**DOI:** 10.1039/c6ra22412a

**Published:** 2016-10-25

**Authors:** Johnas Eklöf, Tina Gschneidtner, Samuel Lara-Avila, Kim Nygård, Kasper Moth-Poulsen

**Affiliations:** a Department of Chemistry and Chemical Engineering, Chalmers University of Technology, Gothenburg SE-412 96, Sweden. Email: kasper.moth-poulsen@chalmers.se; b Department of Microtechnology and Nanoscience, Chalmers University of Technology, Gothenburg SE-412 96, Sweden; c Department of Chemistry & Molecular Biology, University of Gothenburg, Gothenburg SE-412 96, Sweden

## Abstract

The self-assembly of nanoparticles on substrates is relevant for a variety of applications such as plasmonics, sensing devices and nanometer-sized electronics. We investigate the deposition of 60 nm spherical Au nanoparticles onto silicon dioxide (SiO_2_) substrates by changing the chemical treatment of the substrate and by that altering the surface charge. The deposition is characterized by scanning electron microscopy (SEM). Kelvin probe force microscopy (KPFM) was used to characterize the surface workfunction. The underlying physics involved in the deposition of nanoparticles was described by a model based on Derjaguin–Landau–Verwey–Overbeek (DLVO) theory combined with random sequential adsorption (RSA). The spatial statistical method Ripley's *K*-function was used to verify the DLVO–RSA model (ERSA). The statistical results also showed that the adhered particles exhibit a short-range order at distances below ~300 nm. This method can be used in future research to predict the deposition densities of charged nanoparticles onto charged surfaces.

## Abstract 2

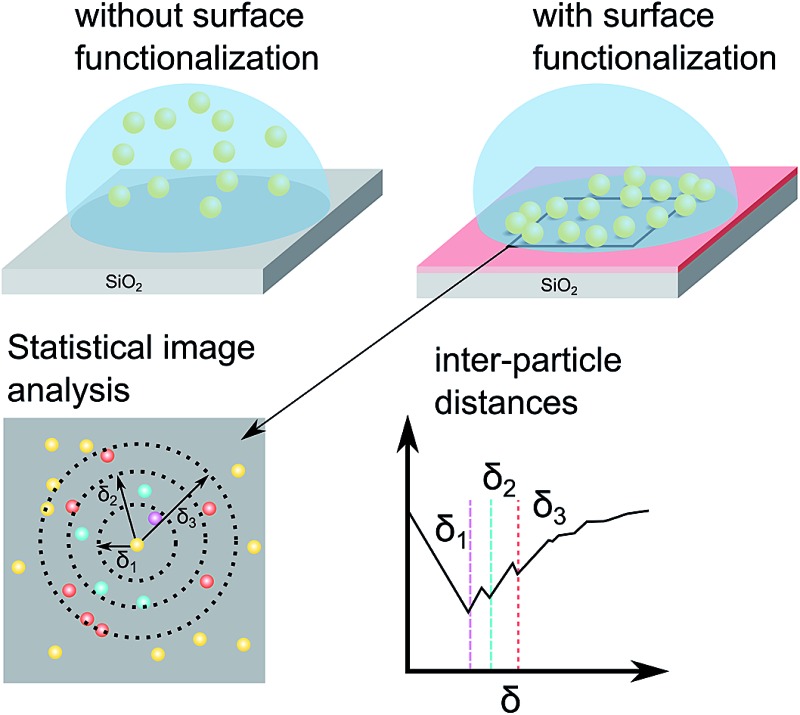
The self-assembly of nanoparticles on substrates is relevant for a variety of applications such as plasmonics, sensing devices and nanometer-sized electronics.

## Introduction

Gold nanoparticles (Au-NPs) are extremely versatile systems whose intrinsic physical and chemical properties can be further enhanced by chemical functionalization to target specific applications. The broad spectrum of Au-NPs applications includes chemical diagnosis,^1,2^ photothermal cancer therapy,^3,4^ plasmonics-assisted sensing,^5,6^ catalysts in epitaxial growth,^7^ catalytic converters,^8^ as antibacterial coatings^9^ as fillers in polymers^10^ and as electrical contacts for the next generation of nanometer-sized electronic devices.^11,12^ A number of these applications necessitate the controlled deposition of Au-NPs from dispersion onto solid state substrates. Gaining control on this process implies a thorough understanding of interactions, namely particle–particle and particle–substrate that occur as particles deposit from dispersions.

Several techniques are available when it comes to the deposition of nanoparticles on substrates. It is for instance possible to deposit uniform nanoparticles in aerosol directly onto a substrate. These techniques require a special setup and it is important that the particles are deposited directly after production.^13,14^ Another possibility is to deposit nanoparticles from colloidal dispersions *via* electrospray deposition and different substrate concentrations have been achieved by changing the deposition time *via* this method.^15^ It is also possible to deposit nanoparticles directly from dispersions by applying an electric potential between *e.g.* a silicon substrate and a Pt/Ir electrode within the colloidal dispersion.^16^ The dispersion of nanoparticles using this method can be varied by changing the time of the deposition. Several reports have shown that it possible to deposit particles *via* convective assembly. In this method a droplet with particles is spread with a glass slide onto a substrate using capillary forces.^17,18^ It has also been shown that it is possible to align the nanoparticles by spreading them on pre-fabricated nano-channels.^19^ Other examples include deposition of nanoparticles by spin-coating a colloidal dispersions on a silicon substrate.^20^ Furthermore another way of depositing nanoparticles if by first depositing a thin film *via e.g.* sputtering or evaporation and then anneal the sample, nanoparticles will formed from the thin film.^21,22^ It should be noted that the evaporation and sputtering techniques requires more advanced instruments and that it might lead to a distribution of sizes of the resulting nanoparticles. The sample must also be tolerant to vacuum and increased temperatures. It is also possible to deposit nanoparticles from solution directly onto surfaces. It is well known that gold form covalent bonds with thiol groups.^23^ This has been utilized when depositing metal and semiconductor nanoparticles on metal surfaces covered with self-assembled monolayers of alkanethiols.^24^ Previous studies have also shown that it is possible to deposit gold nanoparticles onto glass and silicon by treating the surface with organosilanes such as (3-aminopropyl)-triethoxysilane (APTES).^25^

There is a large variety of parameters which are known to affect the density and nearest neighbor distance of deposited nanoparticles, both the characteristics of the nanoparticle dispersion such as concentration of particles, ionic strength, the valency of the ions as well as the size of the particles.^26,27^ In addition stabilizing ligands, surface charge, presence of oxide and temperature can also alter the deposition.^28,29^

The particle–particle interactions and the substrate–particle interactions are believed to be important for the deposition of nanoparticles.^27^ It is known that the densities of citrate stabilized nanoparticles on Si or SiO_2_ are small after deposition,^12^ it is also known that a significant increase in particle density can be observed after different types of activation.^25^ Citrate is a trivalent negatively charged ion, which adsorbs to the nanoparticle surface keeping them suspended in an aqueous dispersion.

In this work we explore the parameter space involved in the deposition of charge-stabilized nanoparticles. We investigate if there is a correlation between surface conditions and the density of particles and nearest neighbor distance after deposition. This was achieved by functionalizing substrates with different chemicals (APTES or poly-l-lysine hydro bromide (PLL-HBr)) as well as different doping of the underlying silicon. The nanoparticles were characterized using scanning electron microscope (SEM) and further analyzed using a statistical image processing software. The substrate surface potentials were investigated using Kelvin force probe microscopy (KPFM) and a physical model to explain the mechanism behind the deposition of the nanoparticles based on Derjaguin–Landau–Verwey–Overbeek (DLVO) theory combined with random sequential adsorption (RSA) was developed. This is also known as the extended random sequential adsorption model (ERSA).

## Experimental

The following products were all ordered from Sigma Aldrich; 99% pure APTES (prod.-nbr. 440140), PLL-HBr (prod.-nbr p7890) with the mol wt of 15 000–30 000 and 60 nm spherical shaped gold nanoparticles (1.9 × 10^10^ NP mL^–1^) (prod.-nbr. 742015) stabilized in sodium citrate. The silicon substrates were both p-doped (boron-doped, from University wafer) and n-doped (phosphorus-doped, from Si-Mat).

All scanning probe measurements were acquired using a Bruker Dimension ICON SPM in peak-force KPFM mode (in air). Scans on a gold, silicon and aluminum grounded reference sample were performed before and after each sample scan to calibrate the contact potential difference measurements. The potentials where extracted in the following way *φ*_sample_ = *φ*_ref_ – *e*(Δ*V*_cpd,tip-ref_ – Δ*V*_cpd,tip-sample_). Here *φ*_ref_ denotes the work function of Au, *φ*_sample_ is the workfunction of the sample, *e* the elementary charge. The contact potential difference Δ*V*_cpd,tip-ref_ is measured on the reference and the contact potential difference Δ*V*_cpd,tip-sample_ is measured on the sample.^30–33^ A PtIr coated Sb n-doped Si SCM-PIT tip with a cantilever with *f*_0_: 60–100 kHz and *k*: 1–5 N m^–1^ were used during the measurements.

The deposited nanoparticles were investigated using SEM. The images were obtained employing the In-lens detector in a Zeiss Supra 60 VP with an accelerating voltage of 12 kV, in a background pressure of 7 × 10^–7^ mbar and with a 30 μm aperture.

Assembly of nanoparticles was enabled by functionalizing SiO_2_ on Si(100) substrates with APTES or PLL-HBr. Both compounds are amine terminated and thus acquire a net positive charge in aqueous solution of neutral pH. The deposition was performed on n and p-doped Si treated in different ways, Si treated with O_2_ plasma and Si activated with either APTES or PLL-HBr.

The nanoparticles were supplied as a colloidal dispersion containing an excess of sodium citrate in order to prevent aggregation. The following method was used in order to decrease the amount of sodium citrate and increase the concentration of nanoparticles. The dispersion was centrifuged for 10 min at 2400 g in a two-step procedure. In the first step two plastic vials (Eppendorf 3810X 1.5 mL) containing 1 mL dispersion each were centrifuged, the supernatant liquid was removed leaving the particles on the bottom of the vial. In the second step the remaining particles were merged in the same vial together with 1 mL of deionized water and centrifuged a second time. The supernatant liquid was removed (100 μL remaining), a droplet of the remaining dispersion was deposited on the substrates for one hour, using a home-built setup with controlled humidity in order to reduce evaporation of the droplet (see Fig. 2). The deposition step was finished by rinsing the substrates with deionized water and blow-dried under a stream of N_2_.

Substrate surface potential characterization was carried out by Kelvin probe force microscopy (FM-KPFM) to reveal the workfunction of bare, O_2_-activated and chemically functionalized SiO_2_ substrates. The deposition of particles was characterized by scanning electron microscope (SEM). The images were analyzed using an image analyzing software using the spatial-statistical method Ripley's *K*-function. The deposition of nanoparticles was also simulated using the extended random sequential adsorption (ERSA) method. The ordinary random sequential adsorption (RSA) method is a Monte-Carlo process which draws particles to a 2D coordinate system.^34^ The deposition of one particle is skipped and moved to a new deposition if the space is already occupied. Two extra steps were added to the ERSA-model, both interactions between particles and interactions between the substrates and the particles were included.

### Substrate activation

**Fig. 1:**
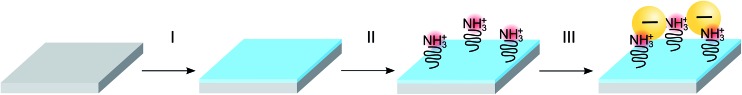
The procedure of activating the SiO_2_ substrates and depositing nanoparticles. The substrate is first treated with O_2_-plasma for 10 seconds which causes a build-up of negative charged hydroxyl groups (I). The substrate is then treated with either (3-aminopropyl)-triethoxysilane (APTES) or poly-l-lysine hydro bromide (PLL-HBr) for 10 min. Subsequently, the deposition of nanoparticles takes place, where the substrate are exposed to a dispersion of citrate stabilized Au-NPs for 60 min. Subsequently, the samples are rinsed with deionized water and blown-dried N_2_ (III).

The silicon substrates used in this work include untreated, O_2_-plasma activated and chemically functionalized Si/SiO_2_ wafers. Both p and n-doped (100) Si chips (10 × 10 mm^2^) were used in this experiment. The n-doped Si was doped with phosphorus and had a resistivity of 1–10 Ω cm. The p-doped Si was doped with boron and had a resistivity of 1–5 mΩ cm. Only native SiO_2_ was present on the substrates, no extra oxide had been grown, the thickness was therefore in the range 3–5 nm. The O_2_-plasma was carried out in a Plasma Therm Batchtop PE/RIE m/95, exposing the substrates for 10 s, seen as step I in Fig. 1, at a power of 50 W an oxygen flow of 10 sccm in an Ar background pressure of 250 mTorr. Chemical functionalization of Si/SiO_2_ substrates was performed using two different molecules respectively, (3-aminopropyl)-triethoxysilane (APTES) or poly-l-lysine hydro bromide (PLL-HBr) (see step II in Fig. 1). For APTES functionalization, an ethanol solution containing 0.0855 mol L^–1^ APTES was drop cast onto a piece of silicon for 10 min. Subsequently, the substrate was immersed into a beaker containing 30 mL ethanol (99%) and stirred for 30 s before immersion into a beaker filled with 30 mL of deionized water and finally blow-dried using a stream of N_2_. The PLL-HBr (0.25 mg mL^–1^, dissolved in water) functionalized substrate was treated in a similar way, where a droplet was put on the Si substrate and kept there for 10 min. The substrate was then rinsed in deionized water and dried with N_2_. Both n- and p-doped Si (100) were treated as mentioned above and used for deposition of nanoparticles.

### Deposition of particles

**Fig. 2:**
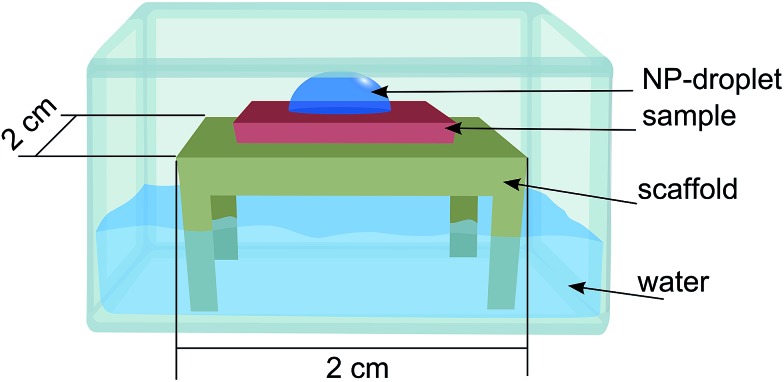
Schematic image of the set-up used for Au-NPs deposition.

The nanoparticle suspension was prepared as described above now containing approximately 3.8 × 10^11^ particles per mL. The suspension was dropped on a substrate of Si/SiO_2_ placed on a substrate holder standing on water inside a petri dish with a lid on (Fig. 2) to saturate the local atmosphere and suppress evaporation of the droplet. The sample was then rinsed in deionized water and dried with N_2_ gas.

### Ripley's *K* and *L* function

SEM images collected over different parts of each substrate were used to study the nanoparticle density and spatial distribution of assembled nanoparticles on the substrate. The inter particle distance of the deposited nanoparticles was estimated by Ripley's *K* function 
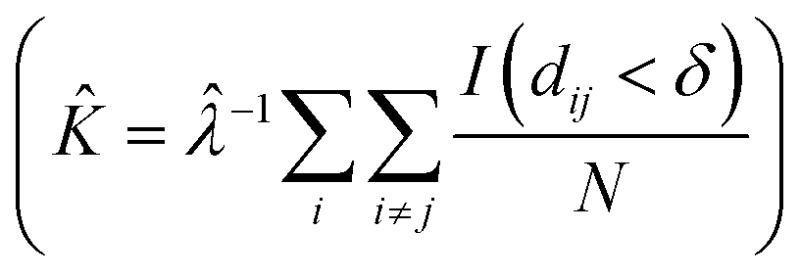
 (ref. 35) a statistical analysis method which describes deviations from spatial homogeneity.

**Fig. 3:**
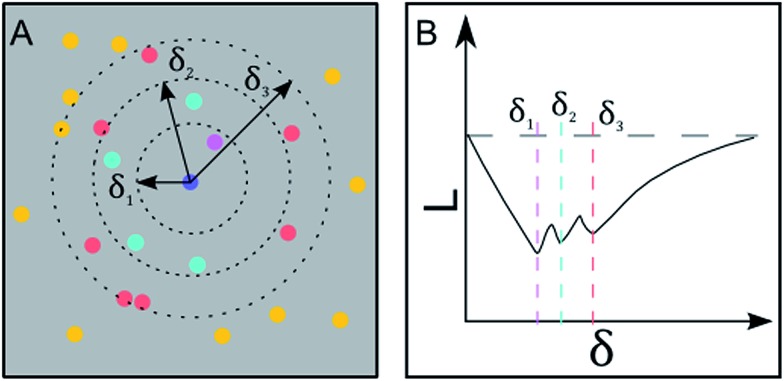
Geometric interpretation of Ripley's *K* function and inter-particle distance detection. (A): The number of particles within a specific radius is summed up. This is done for a set of radii and performed for all particles within a given area. (B): The Ripley's *K* function detects the inter-particle distances as dips in the diagram. Each radius in (A) corresponds to the nearest, second nearest and third nearest neighbor distance.

Here *λ̂* is the estimated particle density in the image (number of particles, *N*, divided by the size of the images), while *I*(*d*_*ij*_ < *δ*) is unity for all points that fulfill the argument (*d*_*ij*_ < *δ*) and zero if not fulfilled. The variable *d*_*ij*_ is the Euclidean distance from one point to all the rest of the points present in the image and *δ* (Fig. 3A) contains a set of limiting distances that grows from one point. This procedure is iterated for all points in the image and the sum of the results is contained in *K*. The data is then treated according to Ripley's *L* function^36^ given by *L* = √(*K*/π) – *δ*.

In this expression, *L* = 0 corresponds to complete spatial randomness. A positive value indicates that the particles are attracted to each other and sit in clusters, while a negative value indicates that the particles on the other hand repel each other until they reach an equilibrium distance.

### Dynamic light scattering (DLS), zeta-potential measurements and pH measurement

The hydrodynamic diameter of the nanoparticles was 74 nm (±1.5 nm), measured by dynamic light scattering (DLS, Malvern Zetasizer Nano) measurements. The error is the standard deviation of ten separate measurements, an extra error is added due to a 2% uncertainty in the machine. It is important to note that the hydrodynamic radius is bigger than the size measured in SEM due to nanoparticle size polydispersity and to solvent molecules and citrate moving along together with the nanoparticles making them appear bigger. A dispersion of nanoparticles was prepared in the same way as described above and placed in the measuring cuvettes. The same dispersion was transferred over to a folded capillary cell equipped with electrodes measuring the zeta potential to –34 mV (±1.6 mV). The pH of the dispersion was determined to be 6.9 using a Jenway model 350 pH meter calibrated with a supplied water solution with pH 4.5.

### Extended random sequential adsorption (ERSA)

The deposition of particles was modelled using the RSA method^37–39^ combined with DLVO theory.^27,40^ In the standard RSA simulation method, particles are randomly positioned on a *xy*-coordinate system one by one, and the particle is rejected if it overlaps any of the existing particles. An extra condition is added to this based on the DLVO theory, where a deposition probability is taken into account. The probability is based on the double layer interaction between the particles and the DLVO interaction between the particle and the substrate. DLVO interaction between two particles consist of two parts, the van der Waals attraction (*W*_vdW_) and the electrical double layer repulsion (*W*_edl_), *W*_tot_ = *W*_vdW_ + *W*_edl_. Ignoring van der Waals interaction, the probability of finding a particle next to another particle at distance *S* (*P*_pp_), we use the Boltzmann distribution: *P*_pp_ = exp(–*W*_edl_/*k*_B_ *T*) with *k*_B_ denoting Boltzmann's constant and *T* the absolute temperature. For small potentials the double-layer repulsion between two particles can be described as^27^ *W*_edl pp_ = 2π*rε*_0_ *ε*_r_ *Ψ*_p_^2^ exp(–*κS*).

Here *Ψ*_p_ is the particle surface potential, *ε*_r_ the relative permittivity of the medium used in the simulation (78.5 for water at room temperature), *r* the radius of the particles, and, *κ* the inverse Debye screening length. The Debye length is set to a maximum of 7 nm, an assumption based on the inter particle distances in Table 1. Note that the previous equation is valid for asymmetric electrolytes, as in our case.

**Table 1:** Inter-particle distance (center-to-center) in nm

	APTES		PLL-HBr	
	Real	Model	Real	Model
p-Doped Si	100 (±10.6)	88.3 (±9.8)	220 (±60.4)	256 (±100.2)
n-Doped Si	92 (±3.5)	85.8 (±10.8)	98.8 (±2.8)	85 (±4.8)
n-Doped plasma treated Si	80.5 (±2.4)	80.8 (±1.5)	122.3 (±19.8)	94 (±26.7)
p-Doped plasma treated Si	106.8 (±2.9)	79.3 (±2.2)	171.3 (±34.3)	161.8 (±53.1)

In order to evaluate the particle–surface adhesion probability, we employ a modified version of *W*_tot_ = *W*_vdW_ + *W*_edl_ that takes into account difference in geometry (plane–sphere instead of sphere–sphere). For low potentials the double layer repulsion between a planar substrate and a spherical particle is described by *W*_edl ps_ = 4π*rε*_0_ *ε*_r_ *Ψ*_p_ *Ψ*_s_ exp(–*κD*) where *Ψ*_s_ is the surface potential of the substrates and *D* the distance between the substrate and the particle's surface. The van der Waals interaction between the substrate and a particle, in turn is given by *W*_vdW_ = –Ar/6*D*.

Here, *A* denotes the Hamaker constant and *r* = 30 nm the particle radius. Using the approximate 
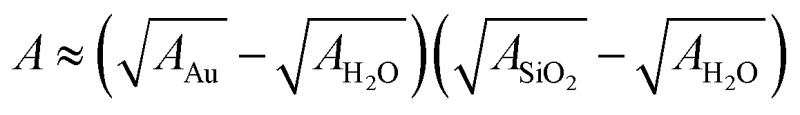
 and tabulated Hamaker constants we obtain the value *A* ≈ 1.5 × 10^–20^ J.^27^

**Fig. 4:**
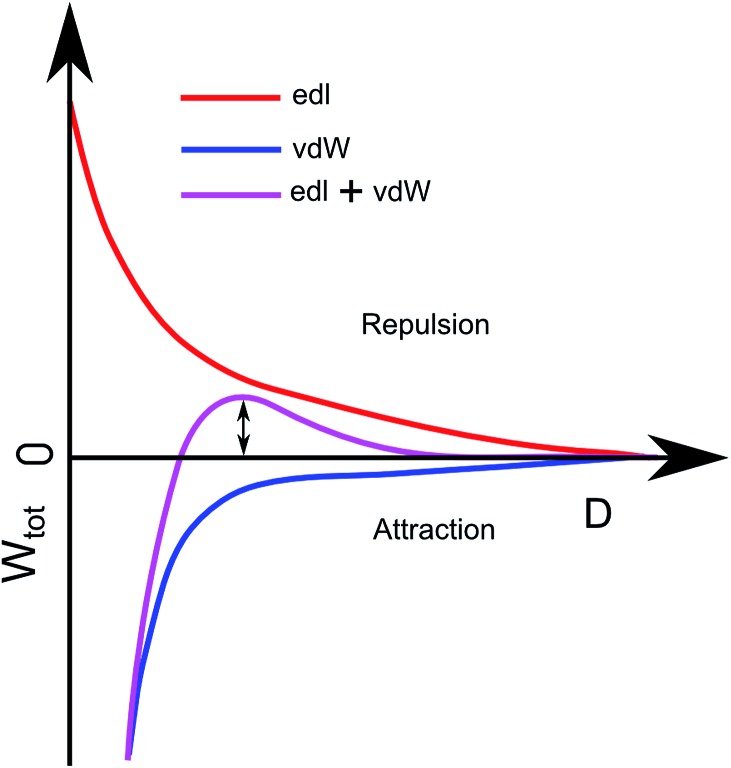
Theoretical description of the interaction between a nanoparticle and the substrate *vs.* the distance between them. The red repulsion comes from the electric double layer and the blue attraction comes from van der Waals interaction. The purple curve is the combined red and blue curve, the energy barrier used for calculating the adhesion probability is indicated with the double arrow.

The combination of *W*_edl ps_ = 4π*rε*_0_ *ε*_r_ *Ψ*_p_ *Ψ*_s_ exp(–*κD*) and *W*_vdW_ = –Ar/6*D* can be seen in Fig. 4 as the purple curve. This plot describes the total interaction between the substrate and a particle. The height of the barrier, Δ*W*, determines the probability for particle adhesion by^41,42^ *P*_sp_ = exp(–Δ*W*/*k*_B_ *T*).

The adhesion probability *P*_sp_ increases as the barrier height Δ*W* decreases and *P*_sp_ is considered equal to one when the barrier becomes negative.

RSA is a Monte-Carlo based process without any real-time dependence. In order to compare our simulations and experimental results, we must calibrate the number of iterations in the ERSA model with the total number of particles that will attempt to deposit on the substrate during a specific time interval, which in turn depends on the diffusion rate of the particles. The number of particles, *N*, that approach the substrate of a specific area within a given time is given by^43–45^ 
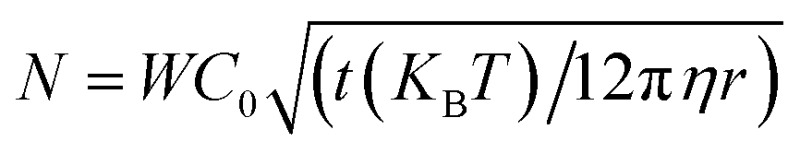
. Here the area in which the model simulates the particles is denoted by *W*, the nanoparticle concentration by *C*_0_ (3.8 × 10^11^ particles per mL), the viscosity of the solvent by *η* (8.9 mg cm^–1^ s^–1^), and the duration of the deposition by *t* (60 min).

## Result and discussion

### SEM images

**Fig. 5:**
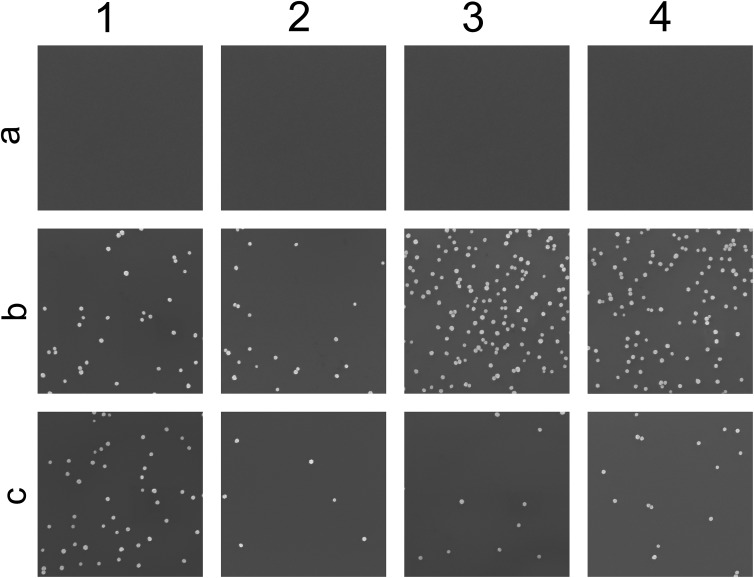
SEM images (2.6 × 2.6 μm^2^) of the substrates after the deposition of nanoparticles. The first row, (a), represents Si(100)/SiO_2_ without any extra activation. The second row, (b), are substrates treated with APTES for 10 min. The third row, (c), substrates treated with PLL-HBr for 10 min. The first column, 1, consists of n-doped Si, the second column, 2, consists of p-doped Si, the third column, 3, consists of n-doped Si treated with 10 s of O_2_-plasma and the fourth column, 4, consists of p-doped Si treated with O_2_-plasma.

12 different substrates (representative SEM images seen in Fig. 5) were treated in different ways. Row a represents Si/SiO_2_ without any extra activation, row b are substrates treated with APTES for 10 min and row c is substrates treated with PLL-HBr for 10 min. The first column represents n-doped Si/SO_2_, column 2 p-doped Si/SiO_2_, column 3 n-doped Si/SiO_2_ treated with O_2_ plasma for 10 s and column 4 consists of p-doped Si/SiO_2_. All samples were exposed to the nanoparticle dispersion for 60 min. In the absence of chemical functionalization, no particles deposited on Si/SiO_2_ (row a, Fig. 5), regardless of substrate doping or O_2_-plasma treatment. This is explained by the clean Si/SiO_2_ substrates acquiring an average net negative charge in water due to deprotonated silanol groups, which causes repulsion of the negatively charged citrate stabilized Au nanoparticles.^46^ Particle assembly is enabled by treating oxidized silicon substrates with either APTES or PLL-HBr, with the adsorption of positively charged amines changing the average net charge of the substrate.

The highest density of deposited particles is found on plasma treated n-doped APTES functionalized Si/SiO_2_ (3b, Fig. 5). APTES form covalent bonds with the deprotonated silanol groups on the surface replacing the negative charge with a positive ammonium group. In the absence of O_2_ plasma treatment, PLL-HBr covered silicon shows the highest density for the n-doped Si (1c, Fig. 5).

### Zeta potential and DLS measurements

The average zeta-potential of the nanoparticles was found to be –34 mV. This value was used as the particles' surface potential in the ERSA-model. The hydrodynamic diameter was 74 nm.

### ERSA model and KFPM measurements

**Fig. 6:**
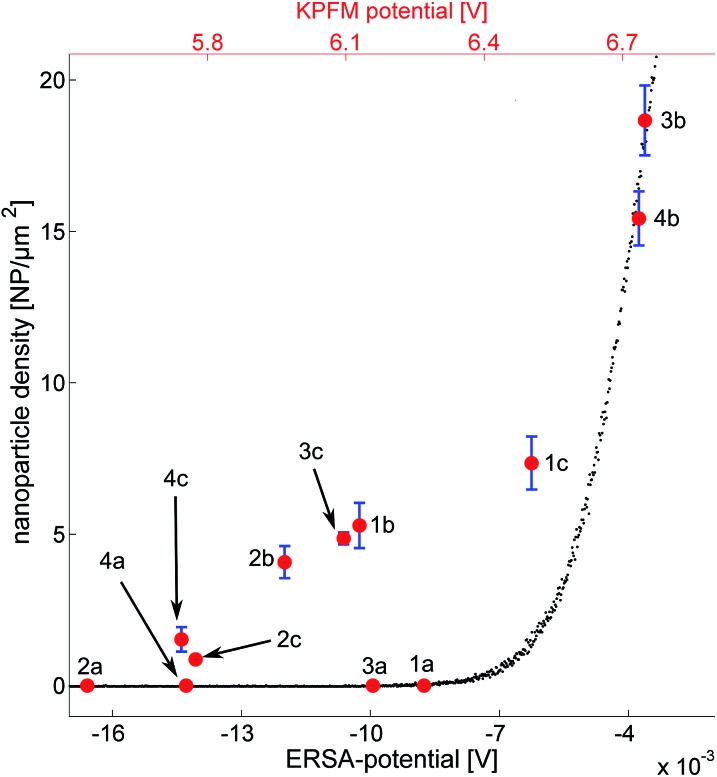
Comparison of ERSA model and experimental results of Au-NPs assembly on Si/SiO_2_ (ERSA potential in the bottom *x*-axis) and the measured KPFM potentials (top *x*-axis). (3b) O_2_ plasma treated n-doped Si activated with APTES. (4b) O_2_ plasma treated p-doped Si activated with APTES. (1c) n-Doped Si activated with PLL-HBr. (1b) n-Doped Si activated with APTES. (3c) O_2_ plasma treated n-doped Si activated with PLL-HBr. (2b) p-Doped Si activated with APTES. (4c) O_2_ plasma treated p-doped Si activated with PLL-HBr. (2c) p-Doped Si activated with PLL-HBr. (1a) n-Doped Si. (3a) O_2_ plasma treated n-doped Si. (4a) O_2_ plasma treated p-doped Si. (2a) p-Doped Si.

The nanoparticle densities as a function of the substrate potential can be seen in Fig. 6. The error is derived from segmenting each image into four sub regions, calculating the density for each region and subsequently the standard deviation and the mean value of those regions. Black data-points represent simulated data which follows an exponential trend. This is expected considering the formulation of the model where the deposition probability is exponentially proportional to the barrier height Δ*W*. The red data points in Fig. 6 represent the measured KPFM potential *vs.* the experimentally observed nanoparticle densities (one hour of deposition). As described above, the substrates treated with first O_2_-plasma and then APTES have the highest density of nanoparticles while the substrates covered with PLL-HBr have smaller densities.

It seems as if the red data points also follow an exponential pattern (except for the clean substrates) just as the ERSA data points do. It would be tempting to explain this with a scaling factor; however, the explanation is probably more complex. The substrate potential extracted from the ERSA model is defined as the potential difference between the surface of the substrate and a point in the solution, where the ion concentration is unaffected by any particle or substrate. The KPFM on the other hand measures the workfunction of the substrate, which corresponds to the work needed to excite an electron from the surface to vacuum.^32^ This could also explain the scaling factor between the ERSA potentials and the KPFM potentials in Fig. 6. It is important to remember that the absolute substrate charge does not need to become positive due to the presence of ammonium groups from APTES and PLL-HBr on the silicon substrate. Instead the substrate charge becomes less negative relative to untreated substrates.

**Fig. 7:**
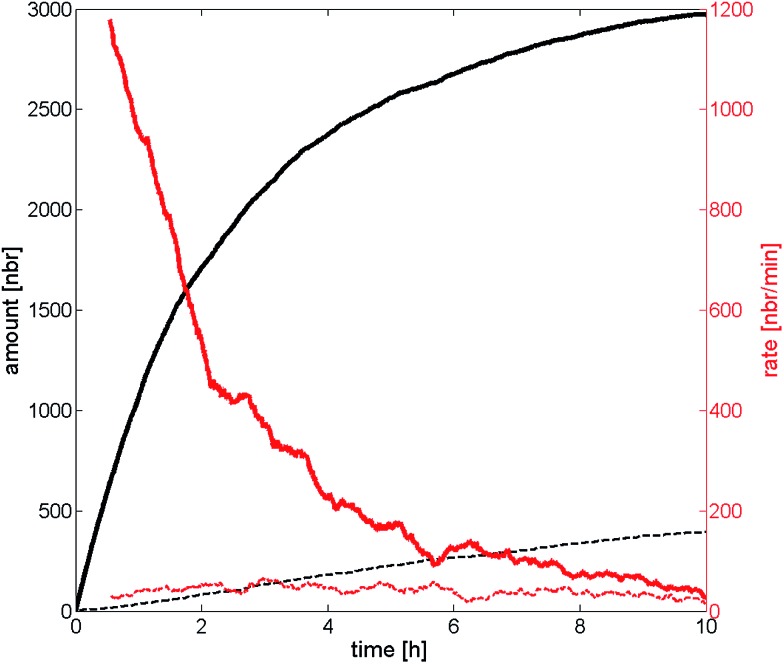
The number of particles deposited after a certain time is represented by the black lines, while the red lines correspond to the deposition rate over time. The solid lines represent high probability of deposition, the dotted lines represents low probability of deposition. The data are collected from the ERSA-model.

The particle adsorption rate is high in the beginning and it quickly decreases for the high probability of deposition case which can be seen in Fig. 7. This indicates that the particles already deposited on the substrate effectively screen the particles remaining in the dispersion in the beginning. The deposition rate has been halved compared to the initial rate already after two hours and the rate is almost zero after 10 hours, approaching the deposition rate of the low probability of deposition case. The deposition rate for the low probability of deposition case has been almost constant throughout the experiment indicating that the screening effect is much lower compared to the other scenario. One interpretation of this behavior is that a substrate with high probability of deposition affects the deposition much more in the beginning, and that the deposited particles play an increasing role the longer the deposition is allowed to run starting by repelling or screening incoming particles. Substrates with a low probability of deposition, on the other hand, play a bigger part throughout the entire experiment and the deposited particles have a much smaller screening effect due to the low number of particles on the substrate. The curves that represent the number of particles on the substrate seem to have a logarithmic behavior (at least in the beginning), indicating that the number would go to infinity when time goes to infinity. This is however not the case since an infinite number of particles on the substrate is not allowed. It would rather go towards a number called the filling factor, which occurs at a coverage of ∼54%.^47^ It should be noted that a substrate with a higher Δ*W* (seen in Fig. 4) will reach the filling factor eventually if the duration of deposition is long enough. The example above is valid for shorter durations, such in our case.

### Statistical analysis

The inter-particle distances (center-to-center) are analyzed in detail using Ripley's *K* function and the results are summarized in Table 1. The error is calculated in the same way here as with the densities in Fig. 6, where the images are segmented into sub regions, each region will have a minima in inter-particle distance and standard deviation and the standard deviation are represented in Table 1. The biggest difference between the real values calculated from SEM images and the ERSA model values are all less than 40 nm (the radius of one nanoparticle), indicating that the ERSA method captures fairly well the physics of the problem. The biggest difference can be seen for the p-doped Si activated with PLL-HBr. For this system the model overestimate the distance between the particles by 36 nm. The model is on the other hand underestimating the distance between the particles for the plasma treated p-doped Si activated with APTES. We attribute these deviations to two effects. First, the particle detection method (recognition of the *x* and *y* coordinates of the particles in a SEM image) cannot distinguish particles placed very close to each other. They will be counted as one particle. The nearest neighbor distance would decrease if also these particles were taken into consideration in the statistical analysis. Second, van der Waals forces are not taken into consideration in the ERSA model, which could explain why the distance is overestimated. The inter-particle distances also show that the particles only affect each other at distances up to ∼300 nm. The pattern therefore displays a short range order but not a long range order.

## Conclusion

We have investigated the deposition of citrate stabilized gold nanoparticles on SiO_2_ substrates. Assembly of nanoparticles on different substrates has been controlled by chemical functionalization of SiO_2_ surface that alters the surface charge density. The focus has been on silicon substrates treated in different ways, including oxygen plasma, followed by adsorption of PLL-HBr or APTES. The position and densities of the nanoparticles on the substrates have been examined by SEM and the properties of the nanoparticles have been confirmed by DLS and zeta-potential measurements. Twelve different substrates where measured with KPFM. These measurements were performed after only surface treatment and not after subsequent deposition of the nanoparticles.

The SEM images of the nanoparticles were analyzed by Ripley's *K* function, in order to retrieve the inter-particle distances after deposition. The experiments demonstrate that the silicon treated with oxygen plasma and APTES has the highest nanoparticle density, whereas silicon without APTES or PLL-HBr shows no nanoparticle deposition. The nanoparticle density *vs.* KPFM potential showed an approximately exponential behavior, with oxygen-plasma-treated and APTES-activated silicon having the highest KPFM values for both n and p doped silicon. A physical model based on random sequential adsorption was also developed, including particle–particle and particle–substrate interactions based on DLVO theory. This ERSA model exhibits an exponential dependence between the particle density and the substrate surface potential, similar to the KPFM data, supporting the hypothesis that the deposition is dependent on the substrate surface potential, which in turn is dependent on the surface treatment, such as oxygen plasma and activation with APTES or PLL-HBr.

The inter particle distances from SEM images are reproduced by the ERSA model, indicating that the particles affect each other over distances shorter than 300 nm and that there is no long-range order. The deposition rate over time also shows that the rate decreases dramatically in the beginning for substrates with high probability of deposition case and that it reaches the same rate as low probability of deposition case substrate after a period of ten hours. This means that the particles quickly start to screen the substrate. The low probability of deposition case substrate on the other hand has an almost constant deposition rate throughout the deposition, showing that the substrate has a greater impact on the particles throughout the entire deposition process. The deposition of nanoparticles is interesting for plasmonic applications and molecular electronics. This model is used to understand the physics behind the deposition of nanoparticles and might be used to predict the time needed to obtain a specific nanoparticle coverage, simplifying future research on nanoparticle deposition.
