# Supplementary material for: Controlling deposition of nanoparticles by tuning surface charge of SiO2 by surface modifications
Source: RSC Adv. 2016 Oct 25;6(106):104246–53. doi: 10.1039/c6ra22412a (PMC5171215; doi:10.1039/c6ra22412a)
Supplement: Supplementary file 1 [file RA-006-C6RA22412A-s001.pdf]

Electronic Supplementary Material (ESI) for RSC Advances.
This journal is © The Royal Society of Chemistry 2016

Supplementary information

Controlling deposition of nanoparticles by tuning surface charge of SiO_2 by surface modifications.

Johnas Eklöf[1], Tina Gschneidtner[1], Samuel Lara-Avila[2], Kim Nygård[3] and Kasper Moth-Poulsen[1]

[1]Department of Chemistry and Chemical Engineering, Chalmers University of Technology, Gothenburg SE-412 96, Sweden.

[2]Department of Microtechnology and Nanoscience, Chalmers University of Technology, Gothenburg SE-412 96, Sweden.

[3]Department of Chemistry & Molecular Biology, University of Gothenburg, Gothenburg SE-412 96, Sweden.

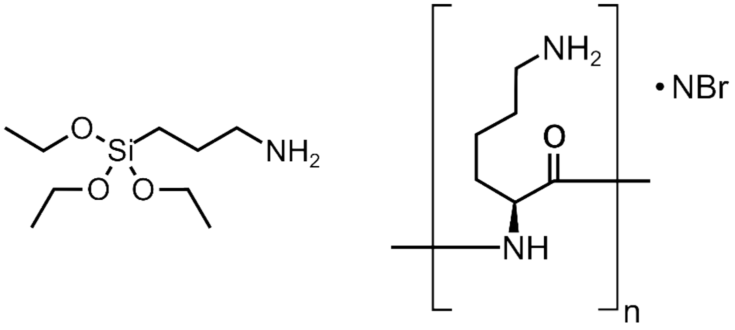

Figure S1: molecules used to chemically functionalize the surfaces. To the left, 3-Aminopropyl)-triethoxysilane (APTES). To the right, poly-L-lysine hydro bromide.

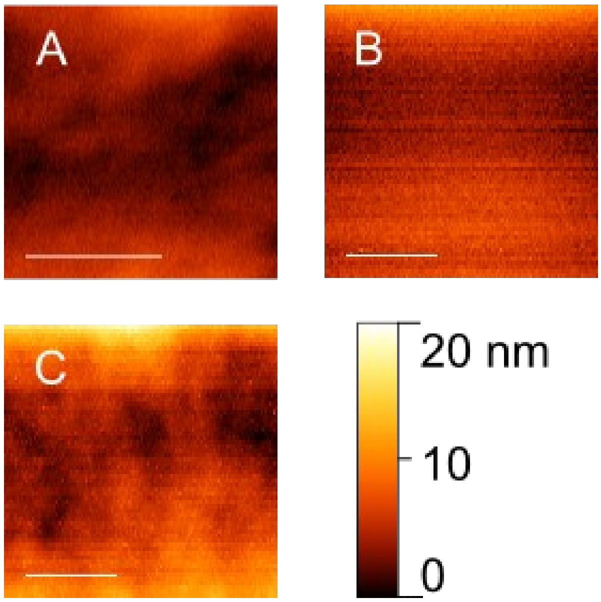

Figure S2: Morphological AFM images. Scale bar is 10 µm A: Si/SiO_2 coated with 3-Aminopropyl)-triethoxysilane (APTES), B: Si/SiO_2 coated with, poly-L-lysine hydro bromide and C: Bare Si/SiO_2.

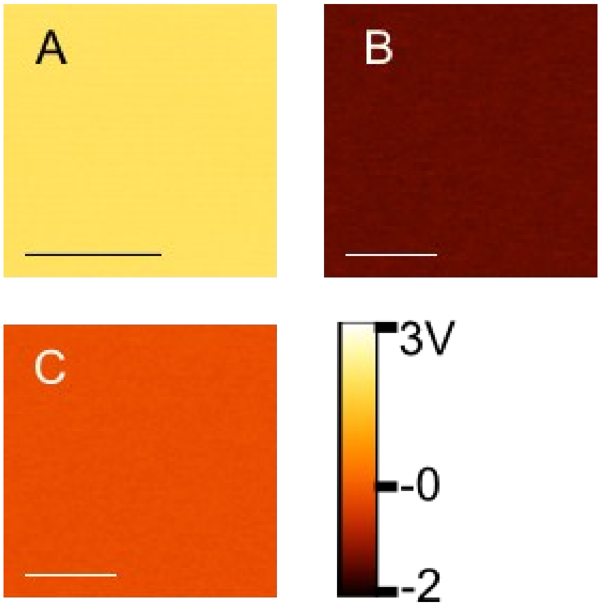

Figure S3: Potential AFM images. Scale bar is 10 µm A: Si/SiO_2 coated with 3-Aminopropyl)-triethoxysilane (APTES), B: Si/SiO_2 coated with, poly-L-lysine hydro bromide and C: Bare Si/SiO_2.

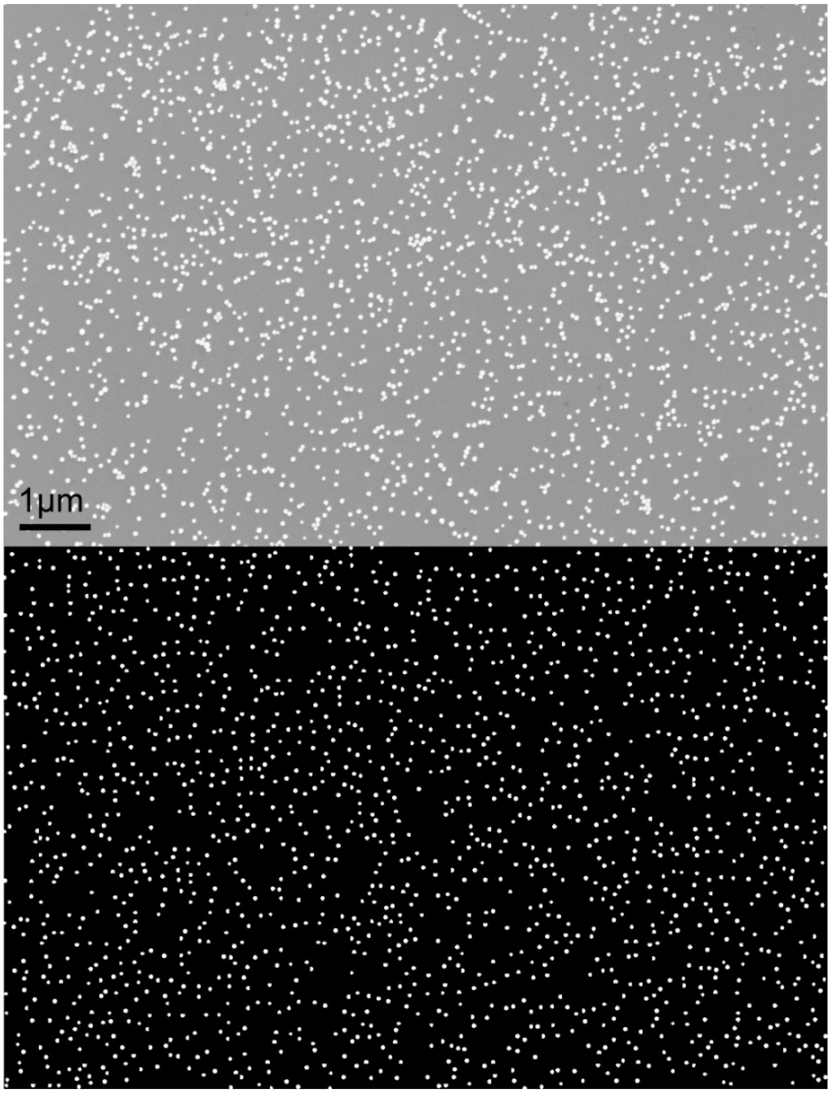

Figure S4: Top picture: SEM over Au nanoparticles deposited on n-doped Si treated with O_2plasma and APTES. Bottom: ERSA modulated nanoparticles with a similar density of nanoparticles. TheL top SEM image was used when calculating the inter particle distance using Ripleys' K function.

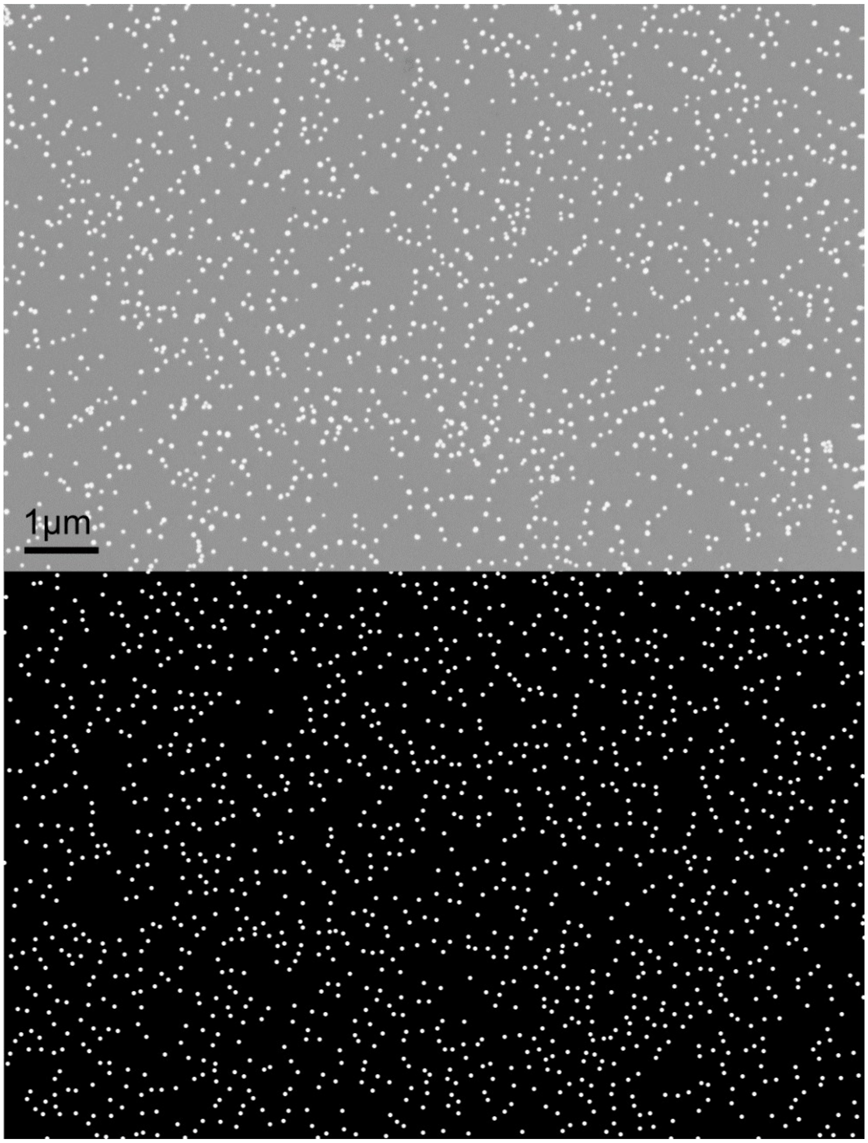

Figure S5: Top picture: SEM over Au nanoparticles deposited on p-doped Si treated with O_2plasma and APTES. Bottom: ERSA modulated nanoparticles with a similar density of nanoparticles. The top SEM image was used when calculating the inter particle distance using Ripleys' K function.

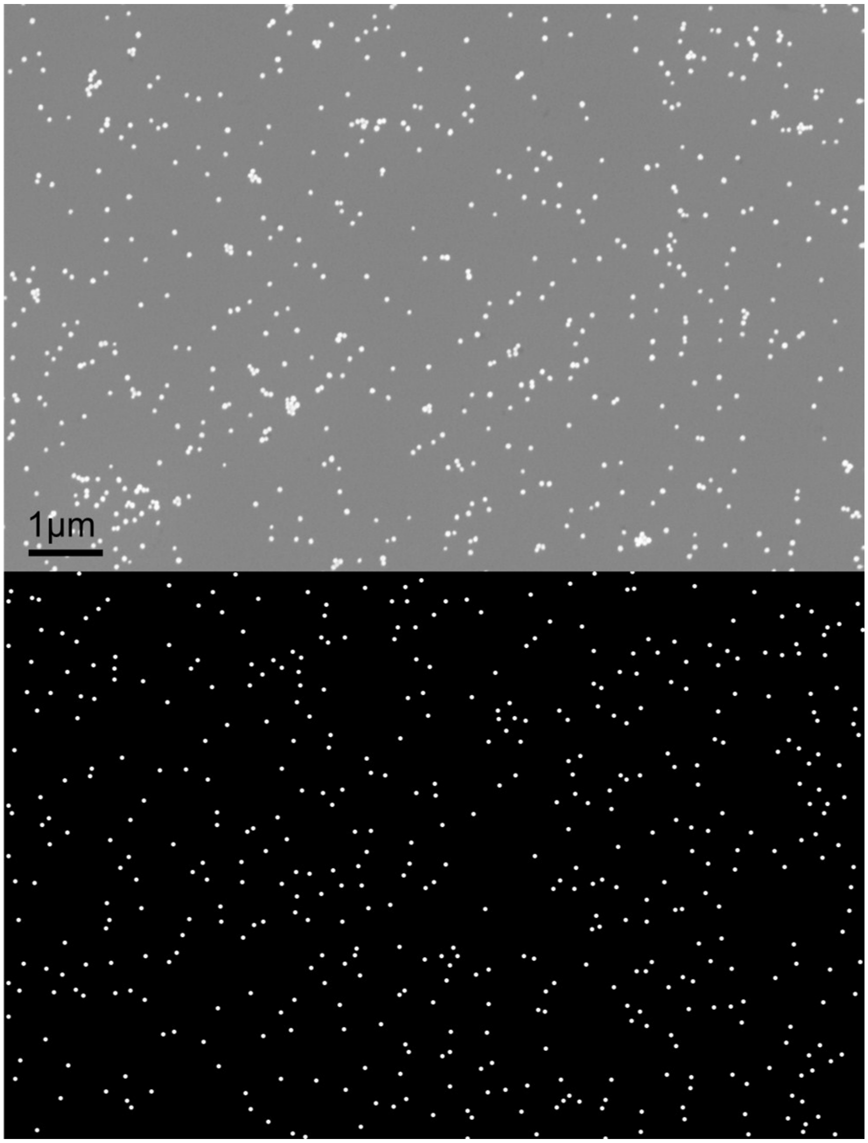

Figure S6: Top picture: SEM over Au nanoparticles deposited on n-doped Si treated with APTES. Bottom: ERSA modulated nanoparticles with a similar density of nanoparticles. The top SEM image was used when calculating the inter particle distance using Ripleys' K function.

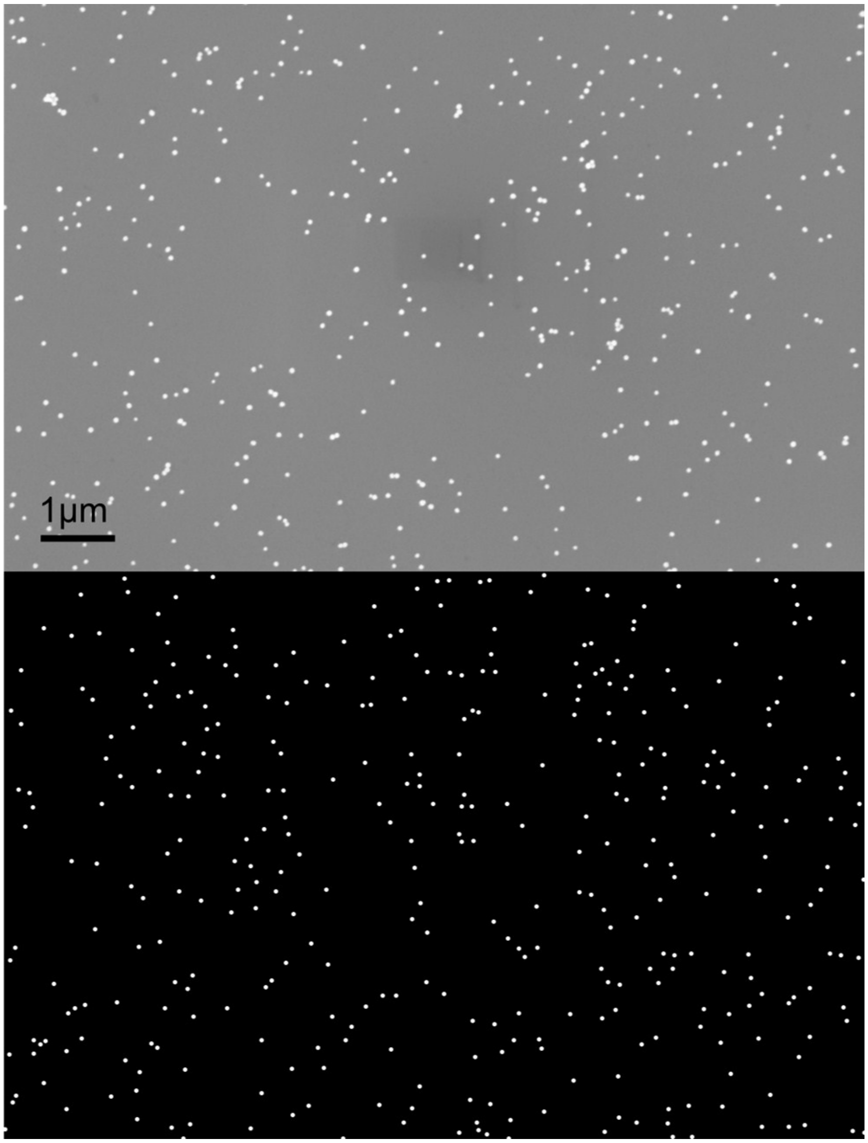

Figure S7: Top picture: SEM over Au nanoparticles deposited on P-doped Si treated with APTES. Bottom: ERSA modulated nanoparticles with a similar density of nanoparticles. The top SEM image was used when calculating the inter particle distance using Ripleys' K function.

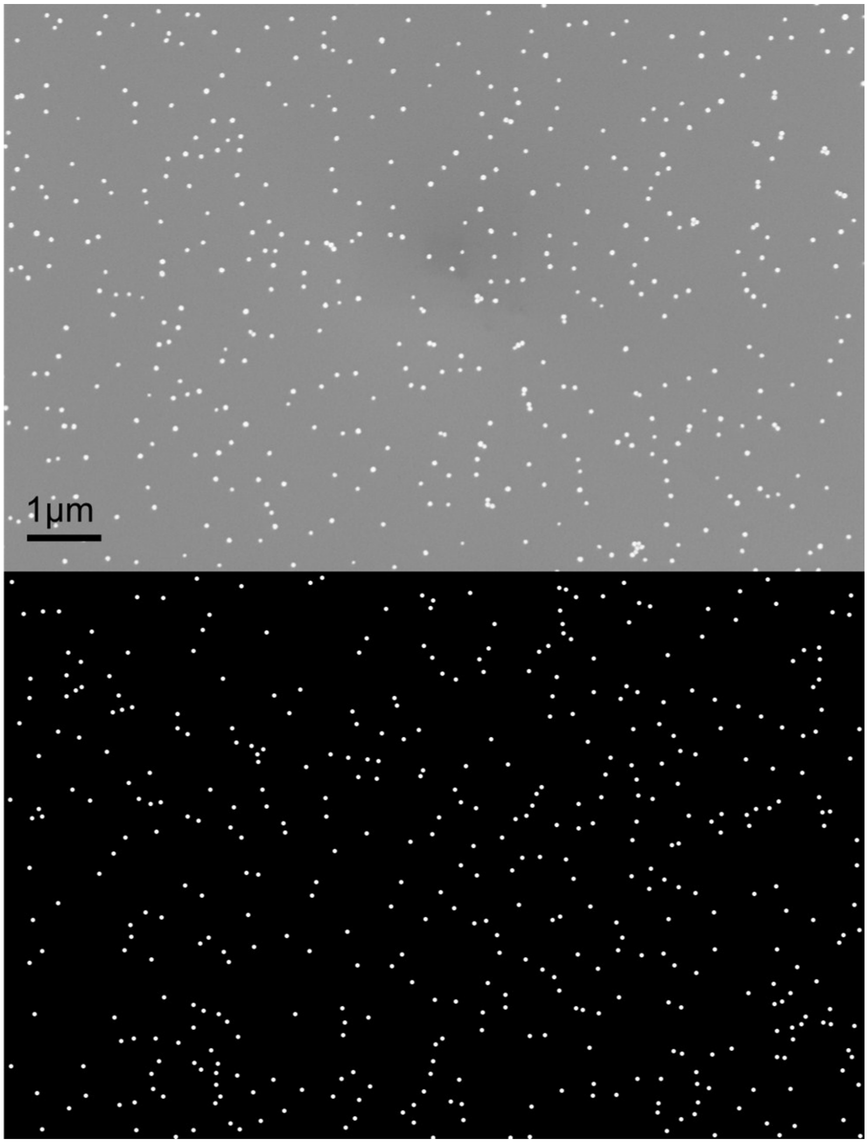

Figure S8: Top picture: SEM over Au nanoparticles deposited on n-doped Si treated with O_2plasma and PLL-HBr. Bottom: ERSA modulated nanoparticles with a similar density of nanoparticles. The top SEM image was used when calculating the inter particle distance using Ripleys’ K function.

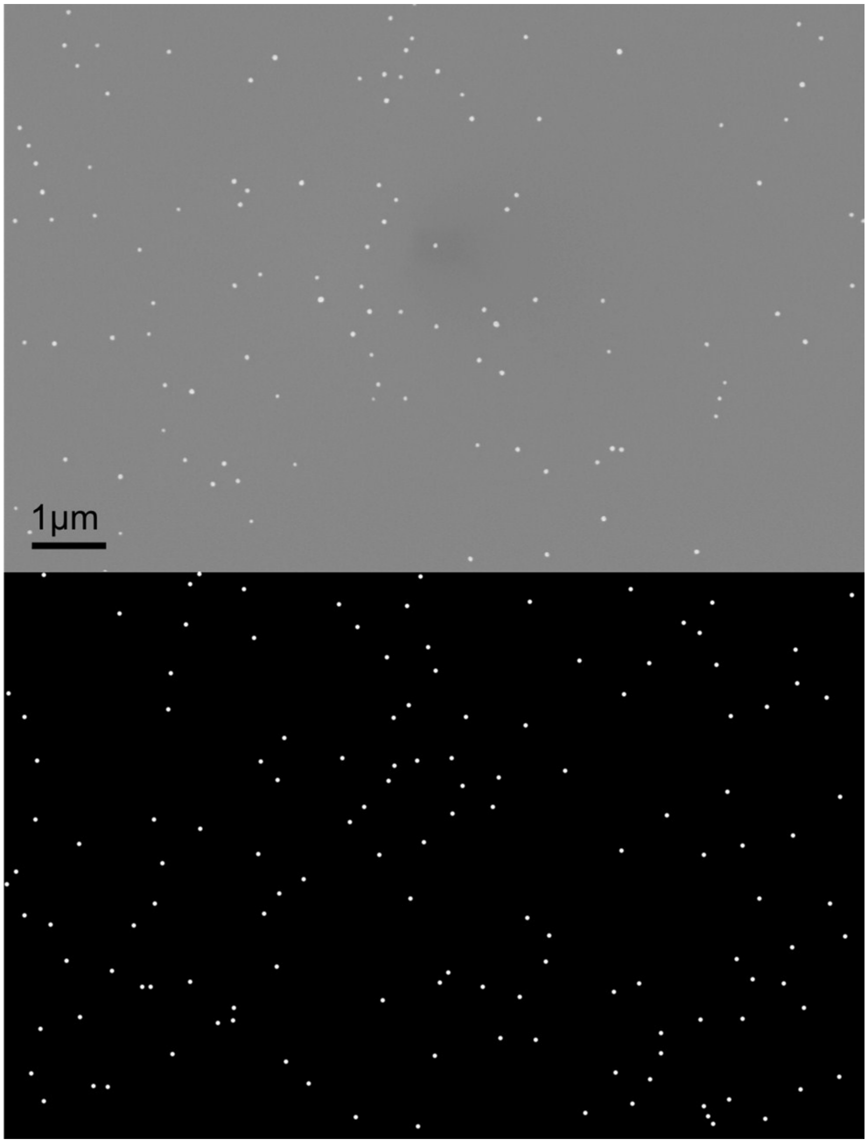

Figure S9: Top picture: SEM over Au nanoparticles deposited on p-doped Si treated with O_2plasma and PLL-HBr. Bottom: ERSA modulated nanoparticles with a similar density of nanoparticles. The top SEM image was used when calculating the inter particle distance using Ripleys' K function.

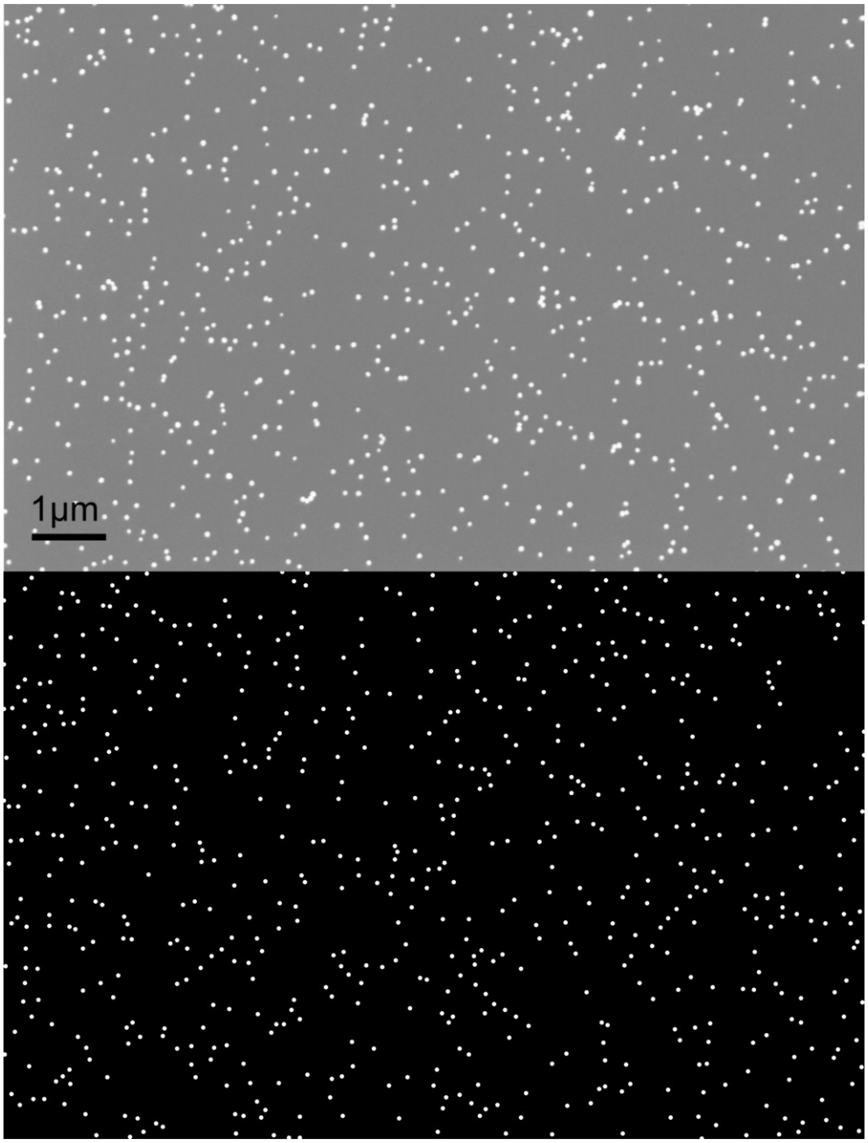

Figure S10: Top picture: SEM over Au nanoparticles deposited on n-doped Si treated with PLL-HBr. Bottom: ERSA modulated nanoparticles with a similar density of nanoparticles. The top SEM image was used when calculating the inter particle distance using Ripleys' K function.

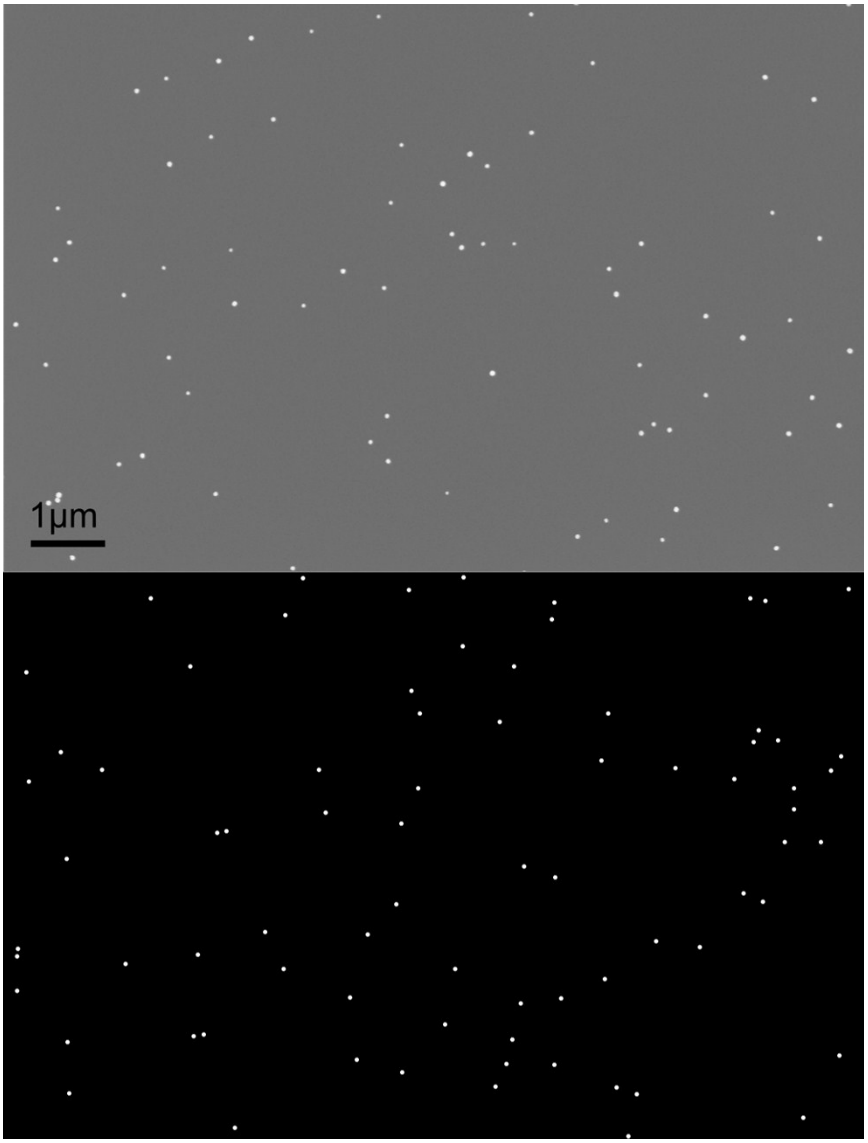

Figure S11: Top picture: SEM over Au nanoparticles deposited on p-doped Si treated with PLL-HBr. Bottom: ERSA modulated nanoparticles with a similar density of nanoparticles. The top SEM image was used when calculating the inter particle distance using Ripleys’ K function.

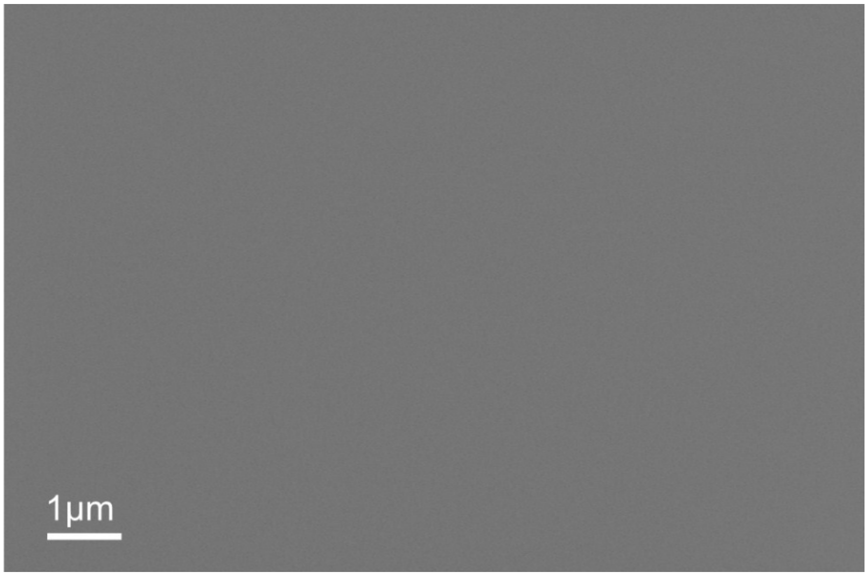

Figure S12: SEM over an attempt of deposit Au nanoparticles on n-doped Si treated with O_2plasma.

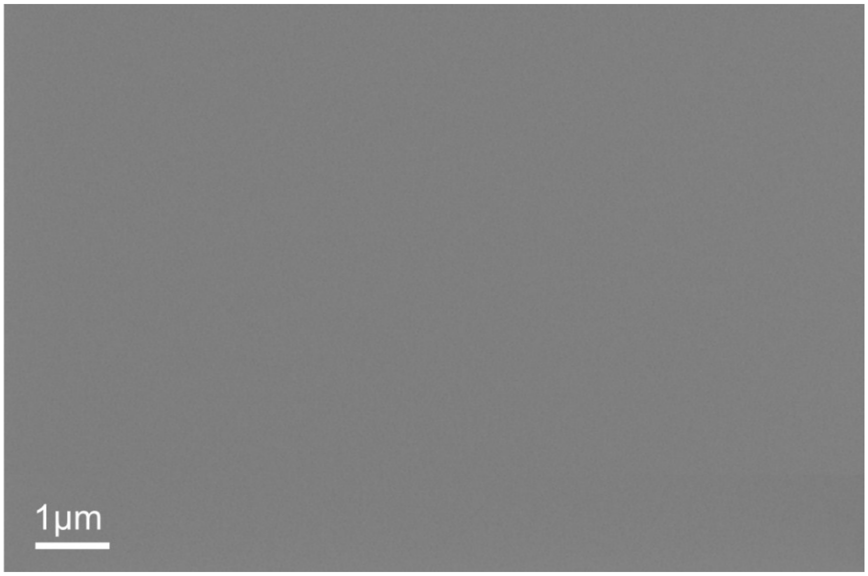

Figure S13: SEM over an attempt of deposit Au nanoparticles on p-doped Si treated with O_2plasma.

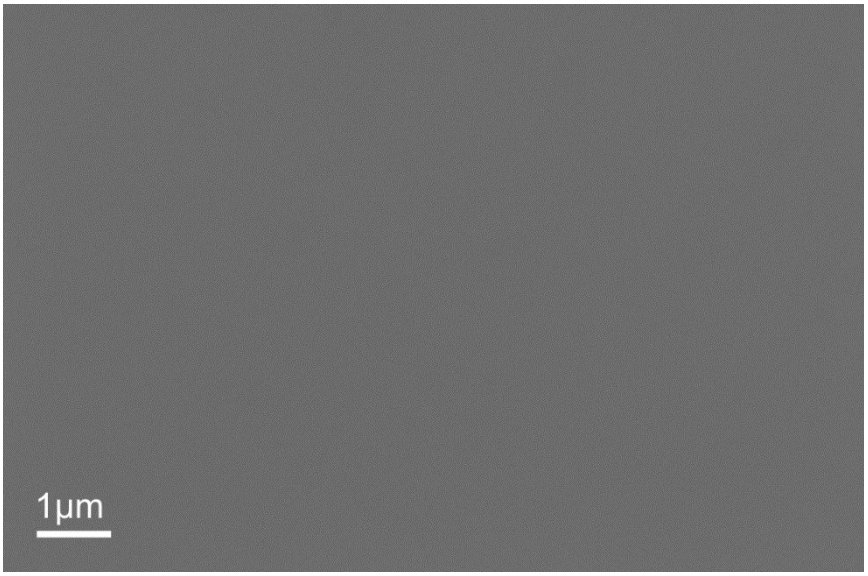

Figure S14: SEM over an attempt of deposit Au nanoparticles on n-doped Si.

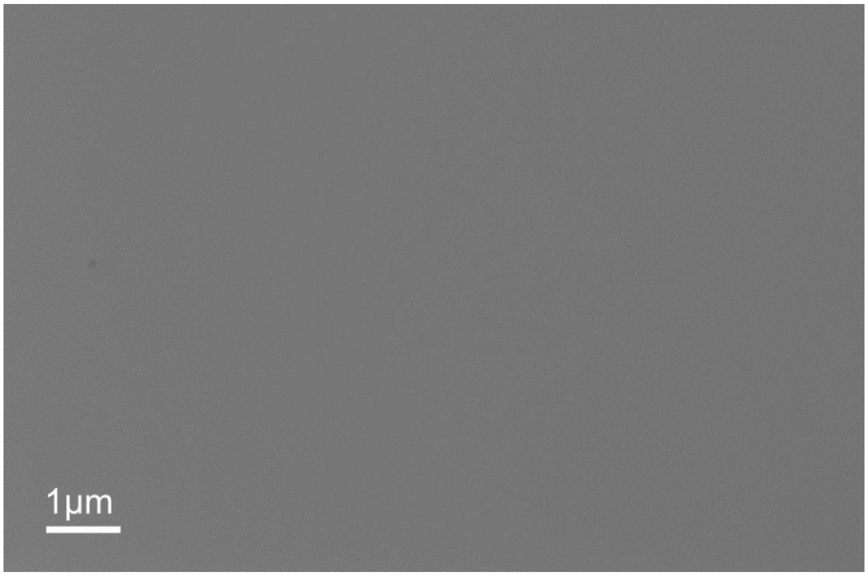

Figure S15: SEM over an attempt of deposit Au nanoparticles on p-doped Si.

Matlab script for modelling of the deposition of nanoparticles

```
 %% time Callibration
% This part calculates how many attempts the Monte-Carlo Process
should try
% to deposit a nanoparticle on the surface, a longer time will
result in
% more nanoparticle deposition attempts
t = 1; % time of deposition in hours
t = t*3600; % time into seconds
kB = 1.38065*(10^-16); % boltzmanskonstant [(g cm^2) / (s^2 K)]
n = 1.9 * 2 * 10^11; % koncentration of nanoparticles [NP / cm^3]
T = 298.15; % ambient temperature[K]
r1 = 30 * 10^-7; % NP radius [cm]
ny = 0.008903; % viscosity of the NP solution [g/cm*s] at T =
298.15[K]
Wsize = 1024*675*(1/88)^2; % simulation area [µm^2]
s_factor = sqrt(1/(12*pi)); %
iter = 10^-8 * (s_factor * n * sqrt( (kB*T)/(ny*r1) )) * sqrt(t) *
Wsize;
iter = round(iter); % The number of deposition attempts
%% probability calculation
% This Sections lists different constants used later in the
calculations
ep0 = 8.854187818*(10^-12); % Vacuum Permitivity [F/m]
epR = 78.5; % Relative Permitivity for the solution
Ah = 1.5*10^-20; % hamaker const [J]
el = 1.60217657*(10^-19); % elementray charge [C]
T = 298.15; % ambient Temperature [K]
r = 30*10^-9; % radius of one NP
nm2pix = 88/1000; % Scaling factors from nm to pix
pix2nm = 1/nm2pix; % Scaling factors from pix to nm
x1 = 0:0.1:120; % Distances between two particles
x = x1*10^-9; % Rescaling into m from nm
xsi_pp = -34 * 10^-3; % Potential of the particles [V]
kappa = 1/(7*10^-9); % 1/ the debye length [1/m]
kB = 1.38065*(10^-23); % Boltzmans constant [m^2*kg/s^2*K]
```

```
%%%%%%% PP probability %%%%%%%%%%%%
% This section describes the probability of finding two particles
next
% two each other
constantsMaster =
struct('ep0',ep0,'epR',epR,'r',r,'xsi_pp',xsi_pp,'kappa',kappa,'kB',
kB,'T',T,'Ah',Ah,'electric_const',el,'nm2pix',nm2pix,'pix2nm',pix2nm
);
% this saves all the constants for later in a struct vector
W_edl_pp = 2*pi*r*ep0*epR*xsi_pp*xsi_pp*exp(-kappa*x);
% this part describes the electric double layer interactions
phi_edl_norm = exp(-W_edl_pp/(kB*T)); % this part normilizes the
phi_edl
x_norm = (x*10^9); % conversion into nm
figure
plot(x_norm,phi_edl_norm,'m.--') % this part plots the probability
of finding two particles next to each other
legend('edl')
%%%%%%%%%%%%%%%%%%%%%%%%%%%%%%%%%%%%%%%%%%%%%%%%%%%%%%%%%%%%%%%%%%%%%%
%%%%%%%
%%%%%%%% SP probability %%%%%%%%%%
% this part calculates the probability of depositing a particle on
the
% surface
x_norm = (x*10^9);
% xsi_sp = linspace(-0.08,-0.06,1000); % the surface potentials used
here
xsi_sp = linspace(-0.0271,-0.011,1000);
totalMAX = zeros(1,length(xsi_sp));
% the for loop, loops each potential and calculates the probability
for
% that potential
```

```
for h = 1:length(xsi_sp)
W_edl_sp = 4*pi*r*ep0*epR*xsi_pp*xsi_sp(h)*exp(-kappa*x);
% This part calculates the electric double layer interactions
between a
% surface and a particle
phi_SP_vdw = (-Ah*r)./(6*x); % this part calculates the van der
waals
%interactions between a surface and a particle
phi_SP_tot = W_edl_sp + phi_SP_vdw;
% this part sums up the electrostatic interactions and the van der
% waals interactions
[maxV,idxV] = max(phi_SP_tot/(kB*T));
totalMAX(h) = exp(-maxV);
% this part takes the high of the barrier and normilizes it
end
figure;
plot(xsi_sp(totalMAX <= 1),totalMAX(totalMAX <= 1),'.')
% plot(xsi_sp,totalMAX,'.')
phi_0_tot = totalMAX(totalMAX <= 1);
xsi_sp = xsi_sp(totalMAX <= 1);
% this part makes sure that we only have probability lower than 1
present
%%
%%%%%%%%%%%%%%%% RSA part simulation
grid = zeros(675,1024); % The size of the deposition area
s = size(grid); % Nbr of elements in size matrix
[x0,y0] = meshgrid(1:s(2),1:s(1)); % meshgrid builds up the
coordinate system
radius = r*nm2pix*10^9; % radius of one NP
count = 0; %
turns = iter; %
maxP = 10000; %
Ptot = zeros(1,turns); %
adsorption = 0; % NBR of adsorbed NP
adsorptionTOT = zeros(1,turns); % -||-
```

```
countTOT = adsorptionTOT; %
xtot = []; % used to save x coordinates
ytot = []; % used to save y coordinates
probTOT = []; %
%%%%%%%%%%%%%%%%%%%%%%%%%%%%%%%%%%%%%%%%%%
% the following part is for saving the values calculated later
yMaster = struct('y',cell(1,length(phi_0_tot)));
xMaster = struct('x',cell(1,length(phi_0_tot)));
adsorptionMaster = struct('adsorption',cell(1,length(phi_0_tot)));
densityMaster = zeros(1,length(phi_0_tot));
probMaster = struct('totalProb',cell(1,length(phi_0_tot)));
phi_0_totMaster = phi_0_tot;
xsi_s_Master = xsi_sp;
constantsMaster;
%%%%%%%%%%%%%%%%%%%%%%%%%%%%%%%%%%%%%%%%%%%%%
runs = 1:length(phi_0_tot);
countMaster = struct('count',runs);
% The following double for loop iterates first through all the
different
% probabilities for the corresponding surface potential,
for h = 1:length(phi_0_tot)
prob_surface = phi_0_tot(h); % the specific surface potential in use
in this iteration
adsorption = 0; % the number keeping track of how many particles
have adsorbed
xtot = []; % xposition of the deposited particles
ytot = []; % yposition -||-
probTOT = []; % the probability of adsorption for each particle
throughout the depotision
grid = zeros(675,1024); % the size of the deposition window, this
keeps track of where the particles have adsorbed
for k = 1:turns; % number of deposition attempts
count = count +1; % count down
x = randi([1 s(2)]); % random x coordinate
```

```
y = randi([1 s(1)]); % random y coordinate
rnd = randi([0 maxP]); % random integear used for the sticking
probability
%%%% new conditions for repulsion between particles
probability = prob_surface; % probability of sticking to the surface
if ~isempty(xtot) % enters if there are other particles on the grid
dist = pdist2([x y], [xtot' ytot']); % calculates the distance to
all other particles to the particle beeing deposited
if min(dist) < 14 % only checks the particle closest to itself
phiTOT = []; % P_sp between each particle near to the particle
trying to deposit
dist_test = dist < 14; %
dist_temp2 = dist(dist_test); % only takes the particles that are
closer than 14
for l = 1:length(dist_temp2) % this loop goes through all the
particles closer than 14 and calculates the probability for each
particle and than calculates the total probability
dist_temp2 = dist_temp2 - 2*radius;
[~,temp] = min(abs(x_norm*nm2pix - dist_temp2(l)));
phi_dist = phi_edl_norm(temp);
phiTOT = [phi_dist phiTOT];
end
phiTOT = [phiTOT prob_surface];
probability = prod(phiTOT);
end
end
Ptot(k) = probability;
circle = (x0 - x).^2 + (y0 - y).^2 <= radius.^2; % draws a
circle/disk/particle
```

```
if grid(circle) == 0 && rnd <= maxP*probability; % puts the particle
on the grid if it is allow according to the probability
grid(circle) = 1;
adsorption = adsorption + 1;
xtot = [xtot x];
ytot = [ytot y];
probTOT = [probability probTOT];
end
adsorptionTOT(k) = adsorption;
countTOT(k) = count;
end
%%%%%%%%%%%% the following code saves the data for later processing
%%%%%%%%%%%
S_xtot = struct('x',xtot);
xMaster(h) = S_xtot;
S_ytot = struct('y',ytot);
yMaster(h) = S_ytot;
S_adsorption = struct('adsorption',adsorptionTOT);
adsorptionMaster(h) = S_adsorption;
adTemp = adsorption/(s(2)*s(1)*((1/88)^2));
densityMaster(h) = adTemp;
S_prob = struct('totalProb',probTOT);
probMaster(h) = S_prob;
end
%%
Master =
struct('constantsMaster',constantsMaster,'xsi_s_Master',xsi_s_Master
,'phi_0_totMaster',phi_0_totMaster,'x_norm_PP',x_norm,'phi_edl_norm_
PP',phi_edl_norm,'xMaster',xMaster,'yMaster',yMaster,'adsorptionMast
er',adsorptionMaster,'densityMaster',densityMaster,'probMaster',prob
Master);
% % %% saving
save('20_hours_loglog.mat','Master')
```
